# Presynaptic 5-HT_2A_-mGlu2/3 Receptor–Receptor Crosstalk in the Prefrontal Cortex: Metamodulation of Glutamate Exocytosis

**DOI:** 10.3390/cells11193035

**Published:** 2022-09-28

**Authors:** Alice Taddeucci, Guendalina Olivero, Alessandra Roggeri, Claudio Milanese, Francesco Paolo Di Giorgio, Massimo Grilli, Mario Marchi, Beatrice Garrone, Anna Pittaluga

**Affiliations:** 1 Department of Pharmacy (DiFar), University of Genoa, Viale Cembrano 4, 16148 Genoa, Italy; 2 Angelini Pharma S.p.A., Viale America 70, 00181 Rome, Italy; 3 IRCCS Ospedale Policlinico San Martino, 16145 Genoa, Italy

**Keywords:** synaptosomes, mGlu2/3 receptor, 5-HT_2A_ receptor, glutamate exocytosis, trazodone, prefrontal cortex, clozapine

## Abstract

The glutamatergic nerve endings of a rat prefrontal cortex (PFc) possess presynaptic 5-HT_2A_ heteroreceptors and mGlu2/3 autoreceptors, whose activation inhibits glutamate exocytosis, and is measured as 15 mM KCl-evoked [^3^H]D-aspartate ([^3^H]D-asp) release (which mimics glutamate exocytosis). The concomitant activation of the two receptors nulls their inhibitory activities, whereas blockade of the 5-HT_2A_ heteroreceptors with MDL11,939 (1 μM) strengthens the inhibitory effect elicited by the mGlu2/3 receptor agonist LY329268 (1 μM). 5-HT_2A_ receptor antagonists (MDL11,939; ketanserin; trazodone) amplify the impact of low (3 nM) LY379268. Clozapine (0.1–10 μM) mimics the 5-HT_2A_ agonist (±) DOI and inhibits the KCl-evoked [^3^H]D-asp overflow in a MDL11,939-dependent fashion, but does not modify the (±) DOI-induced effect. mGlu2 and 5-HT_2A_ proteins do not co-immunoprecipitate from synaptosomal lysates, nor does the incubation of PFc synaptosomes with MDL11,939 (1 μM) or clozapine (10 µM) modify the insertion of mGlu2 subunits in synaptosomal plasma membranes. In conclusion, 5-HT_2A_ and mGlu2/3 receptors colocalize, but do not physically associate, in PFc glutamatergic terminals, where they functionally interact in an antagonist-like fashion to control glutamate exocytosis. The mGlu2/3-5-HT_2A_ metamodulation could be relevant to therapy for central neuropsychiatric disorders, including schizophrenia, but also unveil cellular events accounting for their development, which also influence the responsiveness to drugs regimens.

## 1. Introduction

Several lines of evidence support the role of serotonin [5-hydroxytryptamine, (5-HT)] and glutamate in the pathogenesis of neuropsychiatric disorders, such as schizophrenia [1,2,3]. As far as the schizophrenia is concerned, the involvement of the serotoninergic system has been largely related to central 5-HT_2A_ receptors hyperfunction, as suggested by the psychotomimetic effects of hallucinogenic drugs acting as 5-HT_2A_ receptors agonists, or by of the therapeutic activities of 5-HT_2A_ receptors antagonists and atypical antipsychotics [1,4,5].

The findings from preclinical and clinical studies unveiled that N-methyl-D-aspartate (NMDA) receptors antagonists, such as phencyclidine and ketamine, could induce psychotic symptoms, suggesting that modulation of glutamatergic transmission could be an alternative approach for the treatment of schizophrenia [6]. Consistent with this view, hypofunction of NMDA receptors has been linked to an altered activity of cortical and hippocampal interneurons, resulting in a reduced GABAergic tuning on pyramidal cells, which would lead to central glutamate overactivity. Drugs restoring the GABAergic tuning or, alternatively, reducing glutamate exocytosis may alleviate the symptoms of the disease. Among the glutamatergic ligands displaying antipsychotic activity, agonists of the group II metabotropic glutamate receptors, namely the mGlu2 and mGlu3 receptors, showed antipsychotic-like activity in animal models [7]. These receptors are presynaptic, mainly located on glutamatergic nerve endings and act as inhibitory autoreceptors to reduce glutamate exocytosis, an effect that has been related to their anti-psychotic activity. In addition to presynaptically controlling glutamate exocytosis, mGlu2/3 receptors were also reported to colocalize and functionally crosstalk with 5-HT_2A_ receptors, contributing to an integrated “metamodulation” of synaptic transmission [8,9,10]. This interaction was first described in the mammal cortex, where the 5-HT_2A_ receptors and mGlu2 receptors were shown to physically associate in a heterodimeric complex at the postsynaptic level [11,12,13,14]. The two receptors were proposed to crosstalk in an antagonist fashion, based on the finding that the pharmacological antagonism of mGlu2/3 receptors potentiated the spontaneous excitatory postsynaptic currents elicited by 5-HT_2A_ receptors, whereas mGlu2/3 agonists reduced their expression and functions [15,16,17,18]. The unbalance of the 5-HT_2A_-mGlu2/3 crosstalk was proposed to contribute to the pathogenesis of schizophrenia and drugs recovering the 5-HT_2A_-mGlu2/3 receptor–receptor interaction to physiological levels, which were predicted to be therapeutics for the cure of this disorder.

More recently, we demonstrated that mGlu2/3 and 5-HT_2A_ receptors also physically associate at the presynaptic level in isolated nerve terminals (i.e., synaptosomes) of rat spinal cord, where they functionally interact in an antagonistic fashion to modulate glutamate exocytosis [19,20]. The mGlu2/3-5-HT_2A_ receptors crosstalk was impaired in pathological conditions associated to neuropathic pain, but it recovered by antagonizing the 5-HT_2A_ counterpart of the receptor–receptor complex (i.e., with 5-HT_2A_/antagonists), allowing the conclusion that 5-HT_2A_ antagonists could favor algesia by acting as an Indirect Positive Allosteric Modulator (IPAM) of the mGlu2/3 -mediated signal.

The mGlu2/3 receptors also exist as autoreceptors in cortical glutamatergic nerve endings, where they mainly consist of mGlu2-containing heterodimers [21,22]. These terminals also possess presynaptic inhibitory 5-HT_2A_ receptors controlling the release of glutamate evoked by a depolarizing stimulus [23]. Starting from our background and based on the available data in the literature, this study aimed at investigating whether presynaptic release-regulating mGlu2 autoreceptors and 5-HT_2A_ heteroreceptors coexist and functional crosstalk in glutamatergic nerve endings isolated from the prefrontal cortex (PFc) of rats and to what extent 5-HT_2A_ ligands affect the mGlu2/3 and 5-HT_2A_ receptor crosstalk and the glutamatergic neurotransmission.

## 2. Materials and Methods

### 2.1. Animals

Adult rats (male, strain Sprague Dawley) were obtained from Charles River (Calco, Italy) and were housed in the animal facility of DIFAR, Section of Pharmacology and Toxicology (authorization n° 484 of 8 June 2004). The experimental procedures were approved by the Italian Ministry of Health (authorization n° 875/2017RR), in accordance with the European legislation (CEE, 22 September 2010, no. 2010/63/EU) and with the Italian legislation (L.D. no. 26, 4 March 2014 and 116/1992). In line with the 3Rs rules, all efforts were made to minimize animal suffering and to use the minimal number of animals necessary to produce reliable results.

### 2.2. Preparation of Synaptosomes

Purified synaptosomes were isolated from the rat PFc as previously described [24]. After decapitation, the tissue was rapidly removed and homogenized in 10 volumes of 0.32 M sucrose, buffered with Tris to a pH value of 7.4 (final concentration 0.01 M) using a glass/Teflon tissue grinder (clearance 0.25 mm). The homogenate was centrifuged at 1000× *g* for 5 min to remove nuclei and debris; the supernatant was gently layered on a discontinuous Percoll^®^ gradient (6, 10, and 20% ***v/v*** in Tris-buffered 0.32 M sucrose) and then centrifuged at 33,500× *g* for 6 min. The layer between 10 and 20% Percoll^®^ (synaptosomal fraction) was collected and washed by centrifugation at 19,000× *g* for 15 min.

### 2.3. Experiments of Transmitter Release

PFc synaptosomes were incubated for 15 min a 37 °C in a rotating water bath in the presence of [^3^H]D-aspartate ([^3^H]D-Asp, final concentration: 50 nM)). Identical portions of the synaptosomal suspension were stratified on microporous filters at the bottom of parallel chambers in a Superfusion System and kept at 37 °C [25] (Ugo Basile, Gemonio, Varese, Italy).

After 39 min of superfusion with physiological solution to equilibrate the system, synaptosomes were exposed for 90 s to a high KCl-containing solution (15 mM KCl), in the absence or in the presence of the mGlu2/3 and/or the 5-HT_2A_ receptors ligands. When indicated, 5-HT_2A_ receptors antagonists were added 20 min before agonists. Fractions were collected as follows: two 3-min fractions (basal release), one before (t = 36–39 min, b1), and one after (t = 45–48 min, b3) a 6-min fraction (t = 39–45 min; evoked release, b2). Fractions collected and superfused synaptosomes were measured for radioactivity.

The evoked [^3^H] neurotransmitter release in the absence or in the presence of receptor ligands was expressed as induced overflow and was calculated by subtracting the neurotransmitter content into the first and the third fractions collected (basal release, b1 and b3) from that in the 6 min-fraction collected during and after the depolarization pulse [evoked release, b2; induced overflow = b2 − (b1 + b3)]. When indicated, the effects of receptors ligands were expressed as percent changes versus the 15 mM KCl-evoked tritium overflow.

### 2.4. Western Blot Analysis

PFc synaptosomes were lysed in modified RIPA buffer (10 mM Tris, pH 7.4, 150 mM NaCl, 1 mM EDTA, 0.1% SDS, 1% Triton X-100, protease inhibitors) and the protein content was quantified by using the Pierce™ BCA kit assay (23,225, Thermo Scientific, Waltham, MA, USA). Samples were boiled in SDS-PAGE loading buffer at 95 °C for 5 min and then separated by SDS-10% PAGE (10 or 20 μg/lane) and transferred onto polyvinylidene difluoride (PVDF) membranes (GE10600023, Amersham). Membranes were incubated for 1 h at room temperature in Tris-buffered saline-Tween (t-TBS: 0.02 M Tris, 0.15 M NaCl, and 0.05% Tween 20) containing 5% (*w*/*v*) non-fat dried milk and then probed with rabbit anti-mGlu2 (1:2000, SAB4501318, Sigma Aldrich), mouse anti-5-HT_2A_ (1:200, sc-166775, Santa Cruz Biotechnology, Dallas, TX, USA), and mouse anti-β-actin (1:3000, A5441, Sigma Aldrich) antibodies overnight at 4 °C. After being washed in t-TBS (0.05%), membranes were incubated for 1h at room temperature with appropriate horseradish peroxidase-linked secondary antibodies (1:10000, Sigma-Aldrich, St. Louis, MO, USA). Immunoblots were visualized with an ECL (enhanced chemiluminescence) Western blotting detection system Immobilon Forte Western HRP substrate (WBLUF0100, Merck, Darmstadt, Germany). Images were acquired using the Alliance LD6 images capture system (Uvitec, Cambridge, UK) and analyzed with UVI-1D software (Uvitec).

### 2.5. Biotinylation Studies

Changes in the amount of mGlu2 receptor protein in the PFc synaptosomal plasma membranes were evaluated by performing biotinylation and immunoblot analysis [19]. Briefly, synaptosomes were divided into three aliquots and incubated for 20 min at 37 °C under mild shaking in physiological medium (control synaptosomes), in the presence of 1 µM MDL11,939 or 10 µM clozapine. Synaptosomes (control and treated) were then labelled for 1 h at 4 °C with EZ-Link Sulfo-NHS-SS-Biotin (2 mg/mL; 21,331,ThermoFisher Scientific, Waltham, MA, USA) in PBS/Ca^2+^-Mg^2+^ with the following composition (mM): 138 NaCl, 2.7 KCl, 1.8 KH_2_PO_4_, 10 Na_2_HPO_4_, 1.5 MgCl_2_, 0.2 CaCl2, pH 7.4. The biotinylation reaction was stopped by incubating synaptosomes with PBS/Ca^2+^-Mg^2+^ with 100 mM glycine for 15 min at 4 °C. After two washes in PBS/Ca^2+^-Mg^2^, synaptosomes were lysed in modified RIPA buffer. Samples (100 μg) were then incubated with Dynabeads MyOne Streptavidin T1 beads (65,601; Invitrogen, ThermoFisher Scientific) for 30 min at room temperature under shaking. Beads were added to the biotinylated synaptosomes to pull down the biotinylated proteins, as well as to non-biotinylated synaptosomes, to check the specificity of streptavidin pulldown. After extensive washes, samples were boiled for 5 min at 95 °C in SDS-PAGE loading buffer to isolate the biotinylated proteins from the beads. Eluted fractions were analyzed through immunoblot assay (see Western blot section). The immunoreactivity of mGlu2 receptors was monitored in the total lysate (L), in control (C), in MDL11,939-treated and clozapine-treated biotinylated synaptosomes, and in the streptavidin pull-down of the non-biotinylated synaptosomal lysate (B).

### 2.6. Co-Immunoprecipitation

Protein A Dynabeads (10001D; Invitrogen, ThermoFisher Scientific) were incubated with rabbit anti-mGlu2 antibody (1:500) or with mouse anti-5-HT_2A_ antibody (1:50, sc-166775) in PBS with 0.02% Tween 20 (t-PBS) for 10 min at room temperature. Synaptosomal lysate or total PFc homogenate in RIPA buffer (100 µg) was added to antibody-bound Protein A Dynabeads (I.P.), as well as to the beads without the antibody (negative control, B). After an incubation for 25 min at room temperature under shaking, beads were washed three times in t-PBS and then resuspended in SDS-PAGE loading buffer.

Samples were boiled at 95 °C for 5 min to elute proteins from beads and subjected to Western blot analysis (see section Immunoblotting). mGlu 2receptors were detected with rabbit anti-mGlu2 antibody (1:2000) and 5-HT_2A_ receptors were detected with a mouse anti-5-HT_2A_ antibody (1:200) when the immunoprecipitation was for mGlu2 receptor, or with a rabbit anti-5-HT_2A_ antibody (1:200, 24288, Immunostar, Hudson, WI, USA) when immunoprecipitating for 5-HT_2A_ receptor.

### 2.7. Calculations and Statistical Analysis

Multiple comparisons were performed with analysis of variance (ANOVA) followed by Dunnett’s test or Newman Keuls multiple-comparisons test, as appropriate; direct comparisons were executed by Student’s *t*-test. Data were considered significant for *p* < 0.05 at least.

### 2.8. Chemicals

[2,3-^3^H]D-aspartate (specific activity 11.3 Ci/mmol) was from Perkin Elmer (NET581001MC, Boston, MA, USA). LY379268 was purchased from Tocris Bioscience (2453, Bristol, UK). (±)-1-(2,5-Dimethoxy-4-iodophenyl)-2-aminopropane hydrochloride, (±)-2,5-Dimethoxy-4-iodoamphetamine hydrochloride [(±) DOI], MDL11,939, ketamine, and trazodone were purchased from Sigma (Milan, Italy).

## 3. Results

### 3.1. Presynaptic Release-Regulating mGlu2/3 Autoreceptors and 5-HT_2A_ Heteroreceptors in Rat Prefrontal Cortex Glutamatergic Nerve Endings

**Figure 1:**
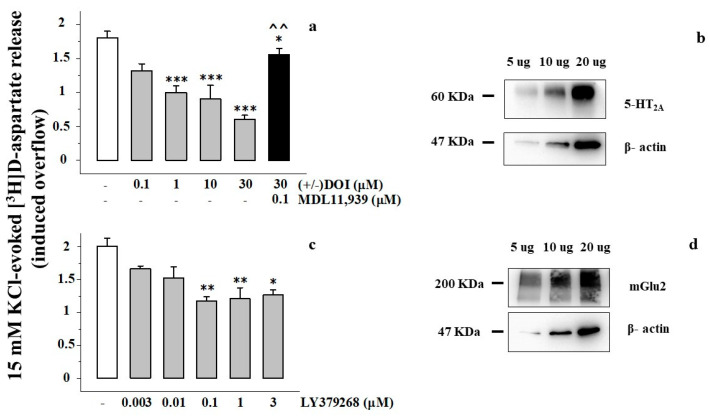
Effects of (±) DOI and of LY379268 on the 15 mM KCl-evoked release of preloaded [^3^H]D-aspartate ([^3^H]D-asp) from synaptosomes isolated from the prefrontal cortex (PFc) of adult rats. (**a**) (±) DOI (0.1–30 μM) inhibits the exocytosis of ([^3^H]D-asp) elicited by 15 mM KCl-enriched medium. Synaptosomes were exposed in superfusion to the depolarizing stimulus for 90 s in the absence or in the presence of the 5-HT_2A_ receptor agonist. When indicated, the 5-HT_2A_ antagonist MDL11,939 (0.1 μM) was added concomitantly to (±) DOI. The release of [^3^H]D-asp in the first 3 min fraction collected (b1) amounted to 0.33 ± 0.02 nCi and represents the 0.90 ± 0.05% of the total tritium synaptosomal content. (**b**) PFc synaptosomes are endowed with the 5-HT_2A_ receptor protein. Synaptosomal lysates were immunoblotted and probed with anti-5-HT2A and anti-β-actin antibodies. (**c**) LY379268 concentration-dependently (0.003–3 μM) reduced the [^3^H]D-asp exocytosis elicited by high K^+^ from synaptosomes isolated from the PFc of adult rats. The release of [^3^H]D-asp in the first 3 min fraction collected (b1) amounted to 0.49 ± 0.06 nCi and and represents the 0.77 ± 0.12% of the total tritium synaptosomal content. (**d**) Western blot analysis confirming the presence of mGlu2 receptor protein in the PFc lysates. Synaptosomal lysates were immunoblotted and probed with anti-mGlu2 and anti-β-actin antibodies. (**a**,**c**) results are expressed as induced overflow; data are the means ± SEM of four to six experiments run in triplicate (three superfusion chambers for each experimental conditions). * *p* < 0.05 vs. respective 15 mM KCl; ** *p* < 0.01 vs. respective 15 mM KCl; *** *p* < 0.001 vs. respective 15 mM KCl; ^^ *p* < 0.01 vs. 15 mM KCl/30 μM (±) DOI. The blots in (**b**,**d**) are representative of 5 to 7 blots run in different days using different samples.

By using an experimental approach that allows for verifying the existence and the role of presynaptic release-regulating receptors in isolated nerve endings (the up-down superfusion of a thin layer of synaptosomes, [26,27] for further details), we confirmed the presence of presynaptic 5-HT_2A_ heteroreceptors in synaptosomes isolated from the PFc of adult rats (Figure 1a,b).

The conclusion relies on the finding that the selective 5-HT_2A_ receptor agonist (±) DOI inhibits the glutamate exocytosis elicited by a mild depolarizing stimulus (Figure 1a). The release of glutamate was quantified as release of preloaded [^3^H]D-asp, a tritiated molecule that mimics glutamate distribution and exocytosis in isolated nerve endings [28,29]. The 5-HT_2A_ agonist concentration-dependently (0.1–30 μM) inhibited the tritium overflow, reaching the maximum effect at 30 μM (Figure 1). The effect of 30 μM (±) DOI was efficiently prevented by the concomitant presence of the 5-HT_2A_ antagonist MDL11,939 (0.1 μM, Figure 1a), as well as by ketanserin (Table 1). Ketanserin (Table 1) and MDL11,939 failed to significantly affect the tritium overflow elicited by the high K^+^ solution (15 mM KCl/100 nM MDL11,939: 1.77 ± 0.08, n = 5; *n.s.*)

The functional observations were supported by the results from Western blot which unveiled the presence of the 5-HT_2A_ proteins in PFc synaptosomes (Figure 1b). An antibody selective for the 5-HT_2A_ receptor subtype unveiled the presence of immunopositivity with a mass consistent with that of the 5-HT_2A_ protein (~60 kDa) in the monomeric form We also confirmed the presence of mGlu2 receptor proteins in rat PFc synaptosomal lysates, which preferentially adopt a dimeric assembly (~200 kDa, Figure 1d). Furthermore, release experiments proved that the mGlu2/3 agonist LY379268 (3 nM to 1 μM) concentration dependently reduced the [^3^H]D-asp overflow when concomitantly added to the depolarizing stimulus, the maximal inhibition being observed when the agonist was added at 0.1–3 μM (Figure 1c). [19,22].

### 3.2. Presynaptic Release-Regulating mGlu2/3 Autoreceptors and 5-HT_2A_ Heteroreceptors Are Functionally Coupled in Rat Prefrontal Cortex Glutamatergic Nerve Endings

**Figure 2:**
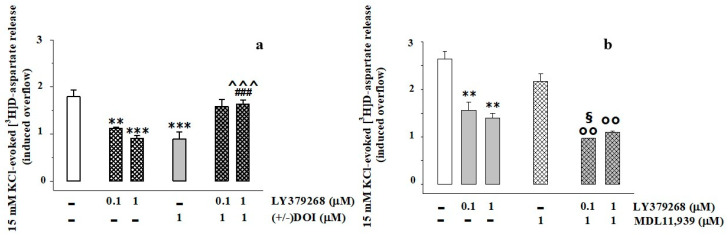
Presynaptic release-regulating mGlu2/3 autoreceptors and 5-HT_2A_ heteroreceptors functionally crosstalk in synaptosomes from the prefrontal cortex of adult rats. (**a**) Effects of (±) DOI (1 μM) and of LY379268 (0.1–1 μM) alone or concomitantly added on the 15 mM KCl-evoked [^3^H]D-asp exocytosis. The release of [^3^H]D-asp in the first 3 min fraction collected (b1) amounted to 0.39 ± 0.02 nCi and represents the 0.88 ± 0.12% of the total tritium synaptosomal content. (**b**) Effects of 1 μM MDL11,939 on the release of tritium elicited by the 15 mM KCl-containing medium in the absence or in the presence of LY379268 (0.1–1 μM). The release of [^3^H]D-asp in the first 3 min fraction collected (b1) amounted to 0.46 ± 0.03 nCi, the 1.03 ± 0.08% of the total tritium synaptosomal content. The results are expressed as induced overflow; data are the means ± SEM of five to six experiments run in triplicate. ** *p* < 0.01 vs. 15 mM KCl; *** *p* < 0.001 vs. 15 mM KCl; ^^^ *p* < 0.01 vs. 15 mM KCl/1 μM LY379268; ### *p* < 0.001 vs. 15 mM KCl/1 μM (±) DOI; °° *p* < 0.01 vs. 15 mM KCl/1 μM MDL11,939; § *p* < 0.05 vs. 15 mM KCl/0.1 μM LY379268.

The inhibitory effects caused by the two receptor agonists [i.e., 1 μM (±) DOI and 0.1–1 μM LY379268] became undetectable when the two agonists were added concomitantly (Figure 2a). Conversely, pre-exposure of synaptosomes to MDL11,939 (1 μM, 20 min before the exposure to the depolarizing stimulus), inactive on its own, amplified the LY379268-mediated inhibition of tritium exocytosis (Figure 2b), causing a significant potentiation of the inhibitory effect when the mGlu2/3 agonist was added at 0.1 μM (Figure 2b).

The impact of 5-HT_2A_ antagonists on the mGlu2/3 receptors was even more evident when synaptosomes were exposed to a low (3 nM), almost inactive, concentration of LY379268. A significant inhibition of the 15 mM KCl-evoked tritium exocytosis was detected in synaptosomes pre-exposed to the 5-HT_2A_ antagonists MDL11,939 (1 μM), ketanserine (0.1 μM), or trazodone (0.1 μM) and then depolarized with 15 mM KCl in the presence of 3 nM LY379268 (Table 2). The antagonists alone, added 20 min before the KC-l stimulus, did not modify the tritium exocytosis (Table 2).

### 3.3. Presynaptic Release-Regulating mGlu2/3 Autoreceptors and 5-HT_2A_ Heteroreceptors Colocalize but Do Not Physically Associate in Rat Prefrontal Cortex Glutamatergic Nerve Endings

**Figure 3:**
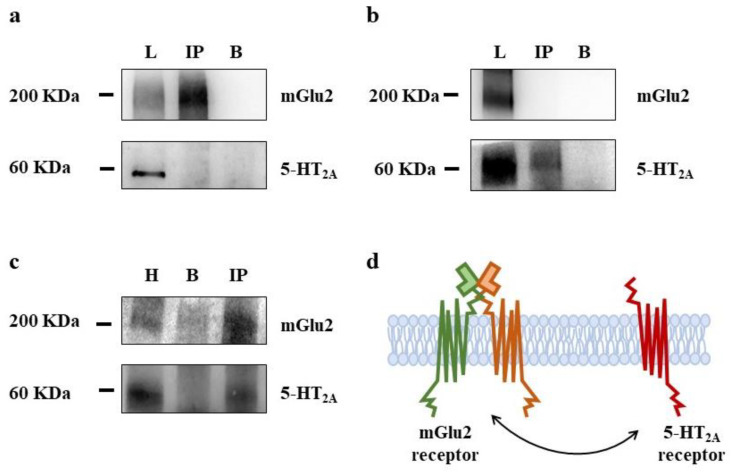
Presynaptic release-regulating mGlu2/3 autoreceptors and 5-HT_2A_ heteroreceptors do not physically interact in synaptosomes from the prefrontal cortex (PFc) of adult rats. mGlu2 receptor proteins (**a**) and 5-HT_2A_ receptor proteins (**b**) were immunoprecipitated from lysates of synaptosomes isolated from the PFc of adult rats. Synaptosomal lysates (L), immuno-precipitates (IP) and negative control (B), here used as a negative control, were analyzed for the presence of mGlu2 and of 5-HT_2A_ immunopositivity. (**c**) mGlu2 receptor proteins were immunoprecipitated (IP) from PFc homogenates and analyzed for the presence of mGlu2 and 5-HT_2A_ receptor proteins (H); negative control (B). The figures in panels (**a**–**c**) are representative of the analysis of five different samples for each experimental condition. (**d**) The cartoon illustrates the colocalization but not the physical association of the mGlu2/3 receptors and the 5-HT2A receptors in PFc synaptosomes.

Immunoprecipitation studies were performed to evaluate whether the 5-HT_2A_ and the mGlu2/3 receptor proteins physically associate in PFc synaptosomes. We focused on the mGlu2 subunit protein since previous studies showed that this is the subunit which preferentially interacts with 5-HT_2A_ receptor proteins by means of specific aminoacid(s) [12,30,31,32]. The lysates of PFc synaptosomes were immunoprecipitated with an antibody recognizing the mGlu2 receptor protein (Figure 3a), or with an antibody recognizing the 5-HT_2A_ receptor protein (Figure 3b). The immunoprecipitates were analyzed for the presence of the mGlu2 and the 5-HT_2A_ receptor proteins.

The anti-mGlu2 immunoprecipitate was immunopositive for the mGlu2 receptor protein, but not for the 5-HT_2A_ protein (Figure 3a). Similarly, the anti-5-HT_2A_ immunoprecipitate from PFc synaptosomes was immunopositive for the 5-HT_2A_ receptor proteins but not for the mGlu2 one (Figure 3b).

Concomitantly, 5-HT_2A_ receptor protein were immunoprecipitated from PFc homogenates and analyzed for the presence of the 5-HT_2A_ and the mGlu2 receptor protein. The immunoprecipitate was positive for both the mGlu2 and the 5-HT_2A_ receptor proteins, well in line with the data in literature [11,12].

### 3.4. Clozapine Inhibits the 15 mM KCl-Evoked [^3^H]D-Aspartate Release in Rat Prefrontal Cortex Glutamatergic Nerve Endings in a MDL11,939-Sensitive Manner

**Figure 4:**
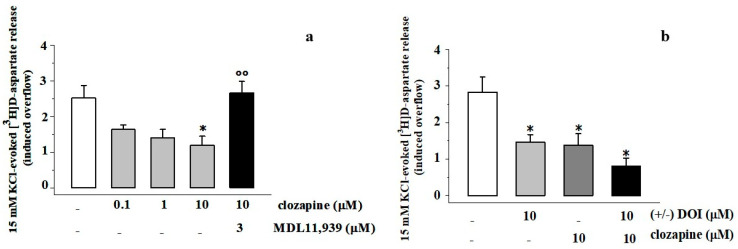
Clozapine inhibits the 15 mM KCl-evoked [^3^H]D-aspartate in synaptosomes from the PFc of adult rats: reversal by MDL11,939. (**a**) Concentration dependent clozapine (0.1–10 μM) inhibits the 15 mM KCl-evoked [^3^H]D-asp exocytosis from PFc synaptosomes in a MDL11,939-dependent fashion. The release of [^3^H]D-asp in the first 3 min fraction collected (b1) amounted to 0.56 ± 0.03 nCi and represents the 1.01 ± 0.06% of the total tritium synaptosomal content. (**b**) Effects of 10 μM (±) DOI and 10 μM clozapine alone or concomitantly added on the 15 mM KCl evoked [^3^H]D-asp exocytosis from prefrontal cortex synaptosomes. The release of [^3^H]D-asp in the first 3 min fraction collected (b1) amounted to 0.57 ± 0.06 nCi and represents the 1.12 ± 0.09% of the total tritium synaptosomal content. Results are expressed as induced overflow; data are the means ± SEM of five to six experiments run in triplicate. * *p* < 0.05 vs. respective 15 mM KCl; °° *p* < 0.001 vs. 15 mM KCl/10 μM clozapine.

Experiments were dedicated to investigate whether and how clozapine can modify the 15 mM KCl-evoked [^3^H]D-asp release from PFc synaptosomes. When concomitantly added to the depolarizing stimulus, clozapine (0.1–10 μM) inhibited the tritium exocytosis elicited by 15 mM KCl in a concentration-dependent, MDL11,939-sensitive manner. The inhibitory effect elicited by 10 μM clozapine was largely prevented by the 5-HT_2A_ antagonist (Figure 4a). (±) DOI and clozapine inhibited to a comparable level the 15 mM KCl-induced tritium exocytosis. Furthermore, when concomitantly added to 10 μM (±) DOI, clozapine (10 μM) did not significantly modify the inhibitory effect elicited by the former drug (Figure 4b).

### 3.5. Preincubation of Prefrontal Cortex Synaptosomes with 5-HT_2A_ Receptor Ligands Does Not Modify the Insertion of mGlu2 Receptor Protein in Synaptosomal Plasma Membranes

**Figure 5:**
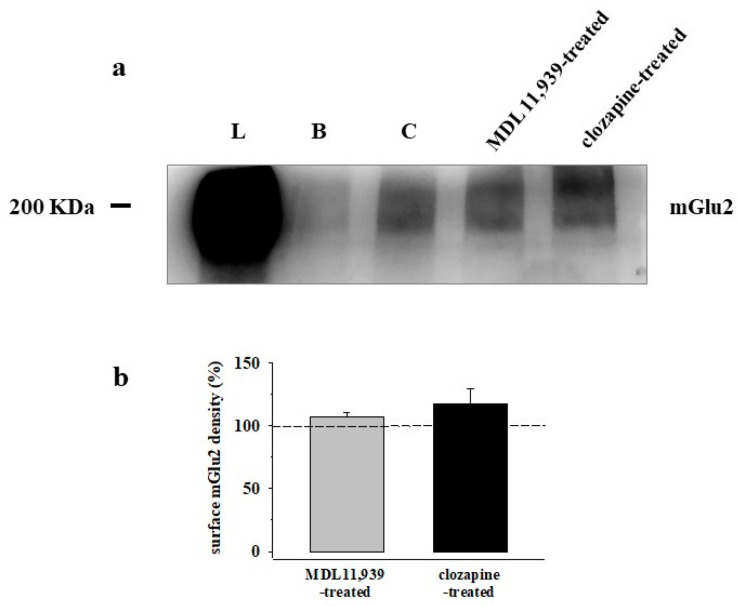
Preincubation of PFc synaptosomes with MDL11,939 or with clozapine does not modify the surface expression of the mGlu2 receptor subunits. (**a**) Representative Western blot analysis of the density of the mGlu2 receptor subunits in PFc synaptosomal plasma membranes following preincubation of synaptosomes with MDL11,939 (1 μM) or with clozapine (10 μM). The blot compares the total synaptosomal lysate (L) and the synaptosomal membranes of particles that were not exposed to biotin, but were subjected to streptavidin pulldown (B), the synaptosomal membranes of untreated particles that were incubated with biotin and then were subjected to streptavidin pulldown (C), the synaptosomal membranes from MDL11,939-treated particles that were incubated with biotin and then were subjected to streptavidin pulldown MDL11,939 (1 μM), and the synaptosomal membranes of clozapine-treated particles that were incubated with biotin and then were subject to streptavidin pulldown (10 μM). The figure in panel (**a**) is representative of five different experiments carried out on different days with samples from different animals. (**b**) Changes in the mGlu2 receptor protein surface density in MDL11,939 (grey bar) and in clozapine (black bar) treated synaptosomal plasma membranes when compared to untreated particles (here indicated as a dotted line). Data are expressed as percent changes versus respective controls.

We asked whether 5-HT_2A_ receptor ligands could affect the insertion of the mGlu2 receptor protein in PFc synaptosomal plasma membranes, as already detected in rat cortical spinal cord synaptosomes [19]. To answer the question, we carried out biotinylation experiments to analyze the surface density of the mGlu2 receptor protein in the plasma membranes from untreated, MDL11,939 (1 μM), or clozapine (10 μM)-treated PFc synaptosomes. The pre-exposure of synaptosomes to the 5-HT_2A_ antagonist did not modify the insertion of the mGlu2 receptor protein in synaptosomal plasma membranes (Figure 5a,b). Similarly, the incubation of synaptosomes with clozapine (10 μM) left unchanged the insertion of the mGlu2 receptor protein in PFc synaptosomal membranes (Figure 5a,b).

## 4. Discussion

5-HT_2A_ receptors are involved in most of the signaling and behaviors induced by psychedelic drugs and represent the targets of several therapeutics to reduce psychosis in patients with neuropsychiatric symptoms [33,34].

Centrally, they preferentially locate in the cortex, the basal ganglia, the cerebellum, and the spinal cord [16,19,35]. In the cortex, they are densely expressed in pyramidal neurons (more than 50% of glutamatergic cells in the rat PFc express 5-HT_2A_ receptors) and to a lesser extent (20–25% on average) in GABAergic interneurons [36,37]. Upon exposure to agonist(s), these receptors preferentially activate G_q/11_ proteins [38], leading to phosphatidylinositol 4,5-bisphosphate hydrolysis and Ca^2+^ release from intracellular stores, which would favor transmitter outflow, as was indeed observed in in vitro microdialysis studies, where 5-HT_2A_ agonists activate excitatory postsynaptic currents [15,39,40,41,42]. In particular, either systemic or intracortical administration of (±) DOI was reported to significantly increase extracellular glutamate levels in the somatosensory cortex of the freely-moving rat, in the absence of concomitant changes in extracellular GABA and glycine levels [43]. However, beside triggering excitatory responses, 5-HT_2A_ receptors also were reported to reduce glutamate exocytosis, and slowing, instead of promoting, the progression of glutamatergic transmission [23,35,40,41,42,44].

The first aim of our study was to shed light on the existence and the functional role of 5-HT_2A_ receptors in controlling glutamate transmission in the PFc. By using the “up-down superfusion of a thin layer of synaptosomes” [26,27], we confirmed the presence of inhibitory 5-HT_2A_ receptors in glutamatergic nerve endings isolated from the prefrontal region of the cortex of adult rats as first proposed by Wang and colleagues in 2006 [23]. The conclusion relies on the findings that (i) PFc synaptosomal lysates are immunopositive for 5-HT_2A_ proteins; (ii) the 5-HT_2A_ agonist (±) DOI efficiently reduces the 15 mM KCl-evoked release of preloaded [^3^H]D-asp; and (iii) the releasing activity elicited by the mild depolarizing stimulus in the presence of the 5-HT_2A_ agonist recovers because of the concomitant presence of the selective 5-HT_2A_ antagonist MDL11,939.

To note, the conclusion is well-consistent with the existence of presynaptic 5-HT_2A_ receptors on thalamo-cortical synapses that control the thalamo-frontal connectivity and the associated cognitive functions [45], but also with the existence of presynaptic 5-HT_2A_ heteroreceptors controlling the glutamate release from layer V pyramidal neurons and modulating conflict anxiety behaviors [46].

These results are best interpreted by assuming that the 5-HT_2A_ receptors in PFc synaptosomes differ from those described by Aghajanian and Marek in 1999 [15] in term of synaptic distribution (the former is located presynaptically in nerve endings specialized for glutamate exocytosis whereas the latter are postsynaptic) and activity (the former inhibit glutamate exocytosis whereas the latter potentiates it).

The functional discrepancy concerning the impact of the receptor on glutamate transmission could be explained by assuming the involvement of different 5-HT_2A_ receptor subtypes, or, alternatively, of a shared 5-HT_2A_ receptor that however couples different G proteins/transducing pathways. Although the first hypothesis cannot be in principle ruled out, the second one is particularly attractive, especially when considering that the shift from facilitation to inhibition of the 5-HT_2A_ receptor-mediated control of glutamatergic transmission emerged when moving from resting to depolarized conditions, which favor the switch of certain release-regulating GPCRs from facilitatory to inhibitory (see for instance the mGlu5 receptors [47]; the CCR5 receptors [48]; the CXCR4 receptors [49]). In general, these receptors promote chemical transmission in resting conditions, but became inhibitory in depolarized conditions, as was indeed observed for the 5-HT_2A_ receptors. The functional shift could be ascribed to the recruitment of different G proteins (G_i_ or G_q_, [12,13]), or alternatively, of different enzymatic pathways, which, in the case of the 5-HT_2A_ receptors, may include GPCR-mediated pathways, β-arrestin-dependent pathways but also G-protein/β-arrestin independent pathways [50].

The second result of the present study confirms the antagonist-like crosstalk linking 5-HT_2A_ heteroreceptors and mGlu2/3 autoreceptors in PFc glutamatergic nerve endings, unveiling however that the receptor–receptor interaction does not imply the physical association of the receptor proteins, as described to occur postsynaptically [11,12,13].

The mGlu2/3 receptors are inhibitory glutamatergic autoreceptors, largely characterized by a pharmacological point of view, that in the cortex of mammals preferentially consist of mGlu2-containing dimers [21,27,51,52]. The results described in the present study show that in PFc glutamatergic synaptosomes, the mGlu2/3 autoreceptors functionally couple the 5-HT_2A_ heteroreceptors as suggested by the finding that (i) their concomitant activation nulls the inhibitory effects elicited by each receptor alone, and, conversely, (ii) the blockade of the 5-HT_2A_ heteroreceptors causes a huge amplification of the mGlu2/3-mediated control of glutamate exocytosis. Notably, the tuning of the mGlu2/3 signal elicited by selective 5-HT_2A_ antagonists (i.e., MDL11, 939, ketanserin, trazodone) was even more pronounced in the presence of low, almost ineffective, concentration of the mGlu2/3 agonist, suggesting that, in PFc glutamatergic nerve endings, 5-HT_2A_ receptor antagonists are fine “metamodulators” of the mGlu2/3 autoreceptors [19,20] and are particularly efficacious in physiological conditions typified by low [glu]_out_ in the synaptic cleft, which cannot elicit an overt activation of the mGlu2/3 autoreceptors. These results in a whole confirm that the two receptors are functionally linked and metamodulate each other in an antagonist manner.

The term “metamodulation” refers to the coexistence and the functional interaction between receptors belonging to different classes [8,9,10,53]. The concept implies that the functional activity(ies) elicited by a drug does not exclusively rely on its binding to the cognate receptor but goes beyond, influencing the functional outcomes triggered by vicinal colocalized receptors, which might belong to different classes, and are activated by other ligands. In this sense, metamodulation implies the colocalization of the two receptors but does not necessarily implicate their physical association.

Notably, as far as the negative interaction linking the 5-HT_2A_ and the mGlu2/3 receptors is concerned, the results from release experiments are consistent with the colocalization of the two receptors on the same terminals (if express in distinct synaptosomal subpopulations, the concomitant activation would cause a larger reduction, corresponding to the sum of the two inhibitory effects) but do not give information on whether they associate in heteromeric complexes [53].

Usually, the physical association of colocalized receptor proteins into heteromeric complexes is evidenced with immunoprecipitation studies (see [11,12] for the 5-HT_2A_-mGlu2/3 receptor complexes) by demonstrating that the immunoprecipitate for one receptor protein is also immunopositive for the “colocalized” protein, which indirectly proves the physical association of the two receptor units.

By using this approach, it was shown that in cortical homogenates from humans and rodents, the 5-HT_2A_ and the mGlu2/3 receptor proteins physically associate in heterocomplexes [11,12,13]. This physical association was confirmed in the present study. 5-HT_2A_ immunoprecipitates from PFc homogenates were immunopositive for either the 5-HT_2A_ and the mGlu2 receptor proteins (Figure 3c). However, differently from the cortical lysates, the 5-HT_2A_ and the mGlu2 protein physical interaction was undetectable in PFc synaptosomal lysates (Figure 3a,b). Either the 5-HT_2A_ or the mGlu2-immunoprecipitate from PFc synaptosomes did not show any immunopositivity for the mGlu2 and the 5-HT_2A_ receptors, respectively, consistent with the conclusion that in these nerve terminals (which are by about 60% glutamatergic in nature), the two receptors merely colocalize.

The finding is particularly intriguing since it suggests that the way the 5-HT_2A_-mGlu2/3 interact each other depends on the synaptic localization of the two receptors (postsynaptic versus presynaptic), but also on the central nervous system (CNS) region under study. Actually, whereas the results of the present study demonstrate that in the PFc the presynaptic 5-HT_2A_ heteroreceptors functionally tune vicinal mGlu2/3 autoreceptors in the absence of physical connection, previous studies demonstrated that the 5-HT_2A_-mGlu2/3 crosstalk in spinal cord glutamatergic nerve endings relies on the physical association of the presynaptic 5-HT_2A_-and mGlu2/3 receptor proteins [19].

The region specificity of the crosstalk linking the 5-HT_2A_ and the mGlu2/3 receptors to metamodulate glutamatergic transmission further emerged when analyzing the ability of one receptor to control the expression and the density of the receptor counterpart in plasma membranes [11,12,13,32]. In spinal cord glutamatergic nerve endings, the antagonism of the 5-HT_2A_ counterpart caused a significant re-distribution of the mGlu2/3 receptor protein in the synaptosomal plasma membranes [19] that, on the contrary, did not emerge in PFc synaptosomes (see Figure 5a,b). These aspects deserve further attention particularly when considering that, besides the physical interaction, are also epigenetic mechanisms of the control of protein expression (particularly of the mGlu2 protein) [54,55]; in addition, some 5-HT_2A_ receptor-dependent phosphorylative processes (which were recently proved to control the phosphorylation of the mGlu2 receptor protein and its coupling to Gi/o signaling [31]) could have a role in 5-HT_2A_-mGlu2/3 receptors crosstalk.

Understanding the events underlying 5-HT_2A_-mGlu2 metamodulation is particularly relevant since, besides its physiological relevance, this receptor–receptor crosstalk might also be involved in the development of central diseases typified by ongoing glutamate overactivity, including schizophrenia, and suggests new targets for innovative therapeutic interventions for the cure of these disorders.

Based on these considerations, it was interesting to analyze the impact on the 5-HT_2A_ heteroreceptors of clozapine, a second-generation atypical antipsychotic which preferentially binds these receptors and that in chronic administration was reported to negatively regulate the density of the mGlu2 receptor subunit [11]. Unlike (±) DOI, which is listed as a full agonist, the pharmacodynamic profile of clozapine at 5-HT_2A_ receptors is far to be fully elucidated. The drug is usually proposed to act as an antagonist or even as an inverse agonist [56,57], but this classification has been questioned by the finding that the chronic administration of clozapine downregulates the central 5-HT_2A_ receptors [58,59,60,61], favoring their internalization [62,63]. Furthermore, clozapine was shown to promote protein kinase B phosphorylation in neurons of the rat PFc [64,65,66], eliciting serotonin-mediated behavior (i.e., the head twitch response) that implies the activation of cortical 5-HT_2A_/_2C_ receptors [67,68]. These observations are best interpreted by assuming that clozapine behaves as a full agonist of the 5-HT_2A_ receptors.

In line with this view, the third main finding of the present study is that acute in vitro clozapine activates the presynaptic 5-HT_2A_ heteroreceptors in PFc glutamatergic synaptosomes, in a MDL11,939-dependent fashion, mimicking (±) DOI in controlling glutamate exocytosis. Furthermore, the inhibitory effects elicited by clozapine and (±) DOI were not additive, nor did clozapine counteract the (±) DOI-mediated inhibition of glutamate overflow, ruling out the possibility that the drug can behave as a partial agonist or even as an inverse agonist at the presynaptic 5-HT_2A_ heteroreceptors under study. Finally, clozapine did not modify the insertion of the mGlu2 receptor proteins in synaptosomal membranes. The last finding is in apparent contrast with data in the literature indicating that the chronic in vivo administration of clozapine downregulates the mGlu2 receptor subunit density ([1] and references therein). Of course, our data relates to the acute in vitro impact of clozapine, which can differ from that elicited by “chronic, in vivo” clozapine (see [7]).

## 5. Conclusions

To conclude, our results demonstrate that presynaptic release-regulating 5-HT_2A_ heteroreceptors and presynaptic release-regulating mGlu2-containing mGlu2/3 autoreceptors colocalize ad functionally couple in glutamatergic nerve endings isolated from the PFc of adult rats. We confirm that the two receptors crosstalk in an antagonist-like fashion, and the 5-HT_2A_ antagonists reinforce the mGlu2-mediated control of glutamate exocytosis. However, differently from what observed in spinal cord nerve endings and in PFc homogenates, the two receptors do not associate in an heteromeric complex, nor do 5-HT_2A_ receptor ligands modify the insertion of the mGlu2 receptor proteins in PFc synaptosomal plasma membranes.

It is important to stress that our results do not in principle exclude the possibility that the 5-HT_2A_ and the mGlu2 receptors could physically interact in glutamatergic neurons in the cortex of mammals, rather they suggest that this event is very limited to the nerve endings (the presynaptic boutons), but is largely diffused in other neuronal regions, including the soma, the axon, the dendritic processes, and the postsynaptic components of the chemical synapses as well.

Finally, we show that clozapine activates the 5-HT_2A_ heteroreceptors in PFc glutamatergic nerve endings in a MDL11,939-sensitive manner, suggesting that the drug could metamodulate the function of the mGlu2/3 autoreceptors in this CNS region.

Our results confirm that 5-HT_2A_ antagonists are IPAM of mGlu2/3 receptors, which could potentially be used in therapy to improve the control of central overt glutamatergicity. Based on the available data and taking into consideration the widespread distribution of these receptors in the CNS, it seems conceivable to propose that the 5-HT_2A_-mGlu2/3 receptors antagonistic-like interaction occurs in different CNS area, with modality and functional outcomes that depend on the region, and which might differently affect the expression and functions of the colocalized receptor(s). As to this aspect, it could be of interest to evaluate whether the 5-HT_2A_-mGlu2/3 receptors crosstalk also occurs presynaptically in the hippocampus, a region which receives a diffuse serotonergic innervation from the raphe and where overt glutamatergicity is a common sign of most of the neurological disorders, including epilepsy and schizophrenia.

## Figures and Tables

**Table 1:** Effects of ketanserin on the release of [^3^H]D-aspartate elicited by 15 mM KCl in the absence or in the presence of (±) DOI.

Stimulus	Induced Overflow (% of Total Tritium Content)	% Changes (vs. KCl-Induced Tritium Overflow)
15 mM KCl	1.73 ± 0.14	
15 mM KCl/100 nM ketanserin	1.65 ± 0.09	−4.62 ± 5.73%
15 mM KCl/10 μM (±) DOI	1.17 ± 0.09 ^a^	−32.36 ± 4.74%
15 mM KCl/10 μM (±) DOI/100 nM ketanserin	1.45 ± 0.11 ^b^	−17.04 ± 5.71%

PFc synaptosomes were exposed in superfusion to 15 mM KCl-enriched medium in the absence or in the presence of (±) DOI (10 μM). When indicated, ketanserin was added concomitantly to the depolarizing stimulus. Results are expressed as KCl-induced overflow (% of total tritium content) and as induced changes with respect to the 15 mM KCl-evoked tritium release. The release of [^3^H]D-asp in the first 3 min fraction collected (b1) amounted to 0.36 ± 0.03 nCi and represents the 0.64 ± 0.06% of the total tritium synaptosomal content. Data are the means of three experiments run in triplicates (three superfusion chambers for each experimental condition). ^a^ *p* < 0.05 vs. 15 mM KCl-evoked tritium overflow; ^b^ *p* < 0.05 vs. 15 mM KCl/10 μM (±) DOI-evoked tritium overflow.

**Table 2:** Effects of 5-HT_2A_ antagonists on the release of [^3^H]D-aspartate elicited by 15 mM KCl in the presence of nanomolar concentration of LY379268.

Stimulus	Induced Overflow (% of Total Tritium Content)	% Changes (vs. KCl-Induced Tritium Overflow)
15 mM KCl	1.77 ± 0.15 (n = 4)	
15 mM KCl/LY379268 (3 nM)	1.57 ± 0.03 (n = 4)	−11.29 ± 9.73%
15 mM KCl/MDL11,939 (1 µM)	1.70 ± 0.09 (n = 4)	−3.76 ± 5.09%
15 mM KCl/LY379268 (3 nM)/MDL11,939 (1 µM)	1.14 ± 0.08 (n = 4)	−35.59 ± 6.32% ^a,b^
15 mM KCl/ketanserin (0.1 µM)	1.69 ± 0.07 (n = 4)	−4.15 ± 4.38%
15 mM KCl/LY379268 (3 nM)/ketanserin (0.1 µM)	1.12 ± 0.06 (n = 4)	−36.72 ± 5.21% ^a,b^
15 mM KCl/trazodone (0.1 µM)	1.74 ± 0.06 (n = 4)	−1.50 ± 3.78% ^a,b^
15 mM KCl/LY379268 (3 nM)/trazodone (0.1 µM)	1.18 ± 0.09 (n = 4)	−33.33 ± 4.35% ^a,b^

Prefrontal cortex synaptosomes were exposed in superfusion to 15 mM KCl-enriched medium in the absence or in the presence of LY379268 (3 nM). When indicated, synaptosomes were exposed for 20 min to 5-HT_2A_ antagonists before the KCl depolarization. Results are expressed as KCl-induced overflow (% of total tritium content) and as induced changes with respect to the 15 mM KCl-evoked tritium release. The release of [^3^H]D-asp in the first 3 min fraction collected (b1) amounted to 0.41 ± 0.02 nCi and represents the 0.65 ± 0.06% of the total tritium synaptosomal content. Data are the means of at least four experiments run in triplicates (three superfusion chambers for each experimental condition). ^a^ *p* < 0.05 vs. 15 mM KCl-evoked tritium overflow), ^b^ *p* < 0.05 vs. 15 mM KCl/3 nM LY379268-evoked tritium overflow.

## Data Availability

Not applicable.
